# Potential Role of Metabolic Intervention in the Management of Advanced Differentiated Thyroid Cancer

**DOI:** 10.3389/fonc.2017.00160

**Published:** 2017-07-25

**Authors:** Sri Harsha Tella, Anuhya Kommalapati, Mary Angelynne Esquivel, Ricardo Correa

**Affiliations:** ^1^Eunice Kennedy Shriver National Institutes of Child Health and Human Development, National Institutes of Health (NICHD/NIH), Bethesda, MD, United States; ^2^Internal Medicine, Washington Hospital Center, Washington, DC, United States; ^3^Endocrinology, Diabetes and Metabolism, Warren Alpert Medical School of Brown University, Providence, RI, United States

**Keywords:** vitamin C, ketogenic diet, metformin, differentiated thyroid cancer, BRAF, KRAS, GLUT1, glutathione

## Abstract

Well-differentiated thyroid carcinoma (DTC) is the most common endocrine malignancy that has an excellent prognosis with a 5-year survival rate of about 98%. However, approximately 50% of the patients with DTC who present with distant metastases (advanced DTC) die from the disease within 5 years of initial diagnosis even after getting the appropriate therapy. Apart from recent advancements in chemotherapy agents, the potential role of metabolic interventions, including the use of metformin, ketogenic diet, and high-dose vitamin C in the management of advanced cancers have been investigated as a less toxic co-adjuvant therapies. The role of vitamin C has been of interest again after a preclinical mice study showed that high-dose vitamin C is selectively lethal to *KRAS* and *BRAF* mutant colorectal cancer cells by targeting the glutathione pathway. This raises the possibility of utilizing high-doses of vitamin C in the treatment of aDTC where *KRAS* and *BRAF* mutations are common. Similarly, alteration of cellular metabolism by low-carbohydrate ketogenic diets can be an important therapeutic strategy to selectively kill cancer cells that mainly survive on glycolysis. Among the potential adjuvant therapies proposed in this paper, metformin is the only agent that has shown benefit in human model of aDTC, the others have shown benefit but in preclinical/animal studies only and need to be further evaluated in large clinical trials. In conclusion, in addition to concurrent chemotherapy options, these metabolic interventions may have a great potential as co-adjuvant therapy in the management of aDTC.

## Introduction

Well-differentiated thyroid carcinoma (DTC) is the most common endocrine malignancy and is classified into three major categories: papillary thyroid carcinoma, follicular thyroid carcinoma, and Hürthle cell carcinoma. The incidence of DTC has grown in recent years due to availability of advanced and sensitive diagnostic tests, which has led to increased detection of micro-carcinomas. DTC has an excellent prognosis with a 5-year survival rate of about 98%. Despite low mortality, this disease has been treated aggressively for fear of more advanced disease and lack of effective tools for risk stratification. These aggressive treatments include high doses of radio-active iodine multiple times that have several side effects on other types of cells and chemotherapy that increase survival but decrease quality of life.

Given its typical protracted course and lack of effective treatments, aDTC can be considered a chronic disease, and safe and low-cost long-term interventions aiming at delaying or prevention of disease progression are more desirable. One such approach could be targeting reprogrammed glucose metabolism of advanced cancers, such as with metformin, ketogenic diet, and high-dose vitamin C (1, 2). These metabolic interventions open new doors with potential role as co-adjuvant therapy in the management of advanced differentiated thyroid malignancies.

## Metformin

Diabetic patients treated with metformin were found to have a lower risk of cancer compared to those who were not on metformin (3, 4). Cancer-related mortality has also been shown to be lower in metformin users when compared to that of non-users (5). Moreover, observational studies in breast cancer have shown that metformin increased sensitivity of breast cancer cells to neoadjuvant therapy (6). A meta-analysis by Cazzaniga et al. (7) concluded that there was a 31% reduction in incidence and mortality from cancer in patients who were on metformin. In DTC patients, a single institutional observational study (8) showed that metformin-treated individuals had smaller tumor size, thus suggesting its potential for tumor growth inhibition. In addition, metformin treatment had also resulted in higher remission rates (8). Based on *in vitro* analysis of DTC cells, *p70S6K/pS6* has been proposed as the most likely molecular target of metformin. By its insulin-sensitizing effect, metformin decreases insulin and insulin growth factor-1 (IGF-1) levels, which are mitogenic hormones. Metformin’s likely role as adjuvant therapy for advanced DTC should be explored further in larger prospective randomized placebo-controlled trials. If proven effective, metformin will be a great adjuvant anticancer agent for advanced DTC (and other cancers) because of its safety and widespread availability.

## Ketogenic Diet

The Warburg effect (9) illustrates the metabolic shift in cancer cells where aerobic respiration is downregulated and glucose is preferentially used to generate energy. The preferential fermentation of glucose to lactate happens regardless of oxygen availability and complete mitochondrial function. Fluro deoxy glucose positron emission tomography scans (FDG-PET scans) utilize this mechanism in the diagnosis of cancer—cancer cells have a selectively enormous amount of glucose uptake compared to normal cells. The shift toward the glycolytic pathway is believed to be mediated by the activation of the insulin/IGF-1-dependent phosphatidylinositol 3-kinase (PI3K)/Akt/mammalian target of the rapamycin (mTOR) system inappropriately, which promotes glucose uptake and trapping by GLUT receptors in cancer cells. Modulation of cellular metabolism by glucose depletion by using ketogenic diets has been proposed as a potential therapeutic strategy to selectively kill cancer cells. Although there is limited data from clinical studies, preclinical studies (10) have shown that the ketogenic diet is a viable option as an adjunct therapy for certain cancers, including advanced DTC.

Ketogenic diets consist of ingestion of very low-carbohydrate, moderate-protein, and high-fat foods, thereby causing preferential metabolism of fats compared to that of carbohydrates and proteins (11, 12). Because of elevated levels of fat-derived ketone bodies and corresponding decreased glucose levels in the blood, alterations in cellular metabolism occur. The ketogenic diet is also shown to reverse redox-signaling pathways that play an important role in tumorigenesis (13). Circulating glucose levels are depleted in the ketogenic diet, thereby compromising glucose metabolism and glucose-related signaling in tumor cells, which in turn results in concomitant reduction of blood insulin and IGF-1 levels, thus leading to downregulation of the PI3K/Akt/mTOR pathway and, in turn, impaired glycolytic metabolism (14). Moreover, in contrast to normal cells, malignant cells are unable to efficiently metabolize ketone bodies. The use of the ketogenic diet opens doors for the management of advanced thyroid cancer in combination with currently available therapies. On the other hand, one must be cautious that the low-carbohydrate diet can amplify cancer cachexia when continued for long periods of time.

## High-Dose Vitamin C

Ascorbic acid (vitamin C) is an essential nutrient and a well-known antioxidant. In 1979, Nobel Prize winners Cameron and Pauling first studied the use of intravenous vitamin C (10 g per day) in 100 patients with various forms of advanced cancer. The results of the study were promising showing an improvement in survival by almost 20 times when compared with the control group (15). However, two other randomized controlled trials (16, 17) failed to replicate these results. It was not until recently that the role of vitamin C has been of interest again after a preclinical mice study (18) showed that high-dose vitamin C is selectively lethal to *KRAS* and *BRAF* mutant colorectal cancer cells by targeting the glutathione (GAPDH) pathway. This raises the possibility of utilizing vitamin C in the treatment of advanced thyroid cancers, where *KRAS* and *BRAF* mutations are common.

Preclinical studies (19) have shown that pharmacologic doses of vitamin C selectively act as a prooxidant in cancer cells thereby increasing the amount of reactive oxygen species (ROS) and exerting antitumorigenic activity. In fact, this prooxidant effect is seen only in tumor cells (19) but not in normal cells. It has been shown that *KRAS* and *BRAF* mutated cancer cells expressed more GLUT1 transporters that had led to increased uptake of vitamin C in its reduced form, dehydroascorbate (DHA). Inside cancer cells, DHA is converted back to vitamin C by glutathione (GSH). During this process, GSH is modified and becomes “oxidized glutathione,” which is further reduced to GSH by nicotinamide adenine dinucleotide phosphate (NADPH). High doses of vitamin C promote activation of ROS, and by this, GSH and NADPH are depleted. As previously alluded to, the presence of GLUT1 receptors on *BRAF* and *KRAS* mutated cells promotes this prooxidant effect of vitamin C in cancer cells. On the contrary, this effect was not seen in *adenomatous polyposis coli* mutated cells, which have less GLUT1 receptors on their cell surface. Interestingly, this lethal effect of high doses of vitamin C on cancer cells is masked in the presence of N-acetylcysteine, which is a potent antioxidant, thereby confirming the pro-oxidative nature of high doses of vitamin C. Hence, tumors that exhibit a high rate of ROS generation coupled with increased GLUT1 expression are likely to benefit from treatment with a high-dose of vitamin C (20). This effect can be taken advantage of and extended to DTC cells that are known to harbor the *KRAS* and *BRAF* mutations. It is important to note that future studies that aim to evaluate the beneficial effects of vitamin C in advanced DTC should use the drug intravenously to achieve adequate concentrations. Two randomized controlled trials (16, 17) failed to show the anticancer benefits of using vitamin C likely due to inadequate vitamin C concentrations as these trials had used the oral forms.

## Conclusion

In summary, promising outcomes of vitamin C described in the management of *KRAS* and *BRAF* mutated colorectal cancer cells opened a new insight into treating aDTC. Similar positive results were seen with metformin and ketogenic diet as discussed above. Thus, we expect that metabolic interventions with high-dose vitamin C, ketogenic diet, and metformin can become an adjuvant therapy for patients with advanced differentiated thyroid cancer. It is interesting to note that all three metabolic interventions exert their effect *via* glycolytic pathways, which are predominantly activated in malignancies (the Warburg effect), particularly in advanced DTC.

These metabolic approaches combined with or without conventional standard therapies have great potential to be beneficial as co-adjuvant therapies in the treatment of advanced DTC. Throughput multicentric randomized trials involving multidisciplinary teams and the use of state-of-the-art technology are substantially needed for advancements in the therapies of aDTC.

## Author Contributions

ST, AK, ME, and RC have made a substantial, direct, and intellectual contribution to the work and approved it for publication.

## Conflict of Interest Statement

The authors declare that the research was conducted in the absence of any commercial or financial relationships that could be construed as a potential conflict of interest.
